# Multi-omics analysis reveals the host-microbe interactions on the dysbiosis of tissue microbiota in male genital lichen sclerosus-induced urethral strictures

**DOI:** 10.1128/spectrum.00074-25

**Published:** 2025-08-18

**Authors:** Zhenwei Yu, Zeyu Wang, Guangyu Mao, Juan Tang, Ruihang Zhang, Lujie Song, Xianjie Xiu

**Affiliations:** 1 Department of Urology, Shanghai Sixth People’s Hospital Affiliated to Shanghai Jiao Tong University School of Medicine 56694, Shanghai, China; 2 Shanghai Eastern Institute of Urologic Reconstruction, Shanghai, China; 3 Department of Pathology, Shanghai Sixth People’s Hospital Affiliated to Shanghai Jiao Tong University School of Medicine 56694, Shanghai, China; College of Life Sciences, Nanchang, Jiangxi, China

**Keywords:** male genital lichen sclerosus, urethral stricture, multi-omics, transcriptome, intratissue microbiome

## Abstract

Male genital lichen sclerosus-induced urethral stricture is a chronic inflammatory disease with significant microbiota dysbiosis. However, dysbiosis inside lesion tissue and its correlation with gene expression in male genital lichen sclerosus (MGLSc) remain elusive. This study investigated the influence of host-microbe interactions on dysbiosis and differential gene expression in MGLSc. Microbiome and transcriptome sequencing were conducted using prepuce samples from 27 MGLSc patients and 17 controls. We also performed immunohistochemistry staining of bacterial markers on prepuce tissue from two cohorts. Furthermore, potential risk factor information available from the MGLSc clinical data was collected and correlated with the differential microbiota. Unclassified Muribaculaceae and *Escherichia coli* were enriched, while *Finegoldia magna*, *Prevotella timonensis*, *Bacillus pumilus*, and *Peptoniphilus harei*, etc., were reduced in MGLSc tissues and decreased in gram-positive bacteria (*P* < 0.05). The functions of differentially expressed genes (DEGs) were associated with immune activation, inflammatory response, innate immunity, and pathogen response. DEGs related to pathogen recognition, such as *TLR1*, *TLR2*, *TLR6*, and *HLA-DOB*, were upregulated (*P* < 0.05). Single-sample gene set enrichment analysis revealed MGLSc lesions enriched immune cells. Clinical correlation analysis indicated that differential microbota was negatively correlated with age (*P* < 0.05) and stricture grade (*P* < 0.05) and was positively correlated with total cholesterol levels (*P* < 0.05), body mass index (*P* < 0.05), and triglyceride levels (*P* < 0.05). Our study provides preliminary clues on host-microbe interactions in MGLSc development, suggesting that tissue dysbiosis may be associated with localized immune dysregulation.

**IMPORTANCE:**

Our study combined full-length 16S rDNA sequencing, transcriptome data, and clinical information from MGLSc patients to explore the relationships between host-microbe interactions and the development of tissue dysbiosis in MGLSc. Importantly, through staining for lipopolysaccharide and lipoteichoic acid, as well as full-length 16S rDNA sequencing, we identified, for the first time, the presence of microorganisms distribution pattern in lichen sclerosus prepuce tissue. Significant differences in the abundance of unclassified Muribaculaceae, *Escherichia coli*, *Finegoldia magna*, and other taxa were observed between the prepuce of MGLSc patients and controls. These differences were associated with altered gene expression in MGLSc patients, while the differential microbiota, in turn, influenced host gene expression. Although patterns of host-microbe interactions varied across populations, dysbiosis was linked to key clinical indicators in MGLSc patients. These findings provide valuable insights into the role of dysbiosis in MGLSc pathogenesis, laying a foundation for understanding disease progression and identifying potential biomarkers.

## INTRODUCTION

Male genital lichen sclerosus (MGLSc) is a chronic inflammatory scarring skin disease (1). Notably, approximately 30% of patients with MGLSc will develop urethral stricture (2). It has also become the major cause of pan-urethral strictures. Male genital lichen sclerosus-induced urethral stricture (MGLSc-US) is more invasive than iatrogenic and traumatic urethral strictures. Its recurrence rate after urethroplasty can be as high as 70% (2). Until now, the exact molecular mechanism of the pathogenesis and involvement of urethral stricture in MGLSc is still unclear. Although not entirely known, a dysregulated inflammatory response is presumed to be one of the underlying mechanisms in MGLSc and MGLSc-US.

Increasing evidence suggests that the local microbiota plays a crucial role in the host immune response. Previous studies, including results from our laboratory, have reported that patients with MGLSc and MGLSc-US exhibit a distinct microbial composition on the skin surface and in the urine compared to healthy individuals and patients with non-MGLSc urethral strictures (3–6). However, previous studies employed balanopreputial sac swabs and urine sampling for microbiota characterization, which precluded analysis of local interactions and their immediate impact on host prepuce expression signatures. To dissect the occurrence and progression mechanisms of MGLSc, more attention needs to be paid to local effects, such as host interactions between microbes within the tissue.

Here, we are first to use transcriptome and 16S rRNA sequencing approaches to identify the core microbiota in MGLSc prepuce lesions and investigate prepuce tissue host-microbe interactions. We hypothesize that prepuce lesions of MGLSc exhibit unique microbial dysbiosis features and that this dysbiosis may demonstrate associations with localized immune dysregulation in the host. We hope this research could broaden our understanding of the MGLSc pathogenesis and potentially lead to the development of microbiome-directed therapies.

## MATERIALS AND METHODS

### Patient selection and sample collection

Patients recruited for this study included adult males clinically diagnosed with MGLSc-US (MGLSc group) and adult males with redundant prepuce (control group) from 2021 to 2023. The study design followed the Strengthening the Reporting of Observational Studies in Epidemiology guideline (Table S1). The clinical diagnosis of MGLSc and control was based on a comprehensive history and examination by highly experienced clinicians. MGLSc diagnosis was based on (i) clinical manifestations, including white plaques, atrophied skin, erythema, erosions, and sclerosis in the anogenital region, and (ii) histopathological findings of presence/absence of hyperkeratosis, thinning/thickening of the epithelium, degeneration of the basal cell layer, dermal collagen homogenization, or lichenoid lymphocytic or plasmacytic infiltrate (7). Control samples derived from urology patients undergoing redundant prepuce surgery exhibited no LS-related clinical and pathological characteristics. Exclusion criteria were (i) acute urinary tract infection; (ii) acute genital skin infection; (iii) history of sexually transmitted diseases (gonorrhea, syphilis, and HIV); (iv) congenital genital anomalies not caused by MGLSc; (v) genital pigmentary disorders not caused by MGLSc; and (vi) phimosis patients. Phimosis serves as a typical clinical sign of MGLSc (8) and also is associated with a higher incidence of urinary tract infections (9). Moreover, uncircumcised phimosis males enriched the sulfate-reducing bacteria with high levels of thiosulfate reductase and polysulfate reductase. These microbial features are associated with hydrogen sulfide-mediated inflammatory responses (10).

The sample size was determined based on previous microbiome studies of lichen sclerosus using skin swabs, which typically included 10–30 participants per group (3, 11–13). Based on strict inclusion and exclusion criteria, foreskin samples were ultimately collected from 27 MGLSc patients (lesional sites) and 17 individuals with redundant prepuce for 16S rDNA sequencing, transcriptome sequencing, and histopathological analysis. The post hoc power analysis was performed using the relative abundance of a representative differentially abundant taxon identified by LEfSe. Based on the current sample size, the statistical power was estimated to be 65%. Details of the power analysis were provided in the Supplementary Materials.

All preputial tissue samples, including both redundant foreskin from control individuals and lesion tissues from MGLSc patients, were collected intraoperatively under strict sterile conditions by the operating surgeon. The obtained foreskin tissues were divided using sterile scalpel blades, with each tissue piece approximately 2 × 3 mm in size. A portion of the excised foreskin tissue was retained for pathological examination. The divided foreskin tissues were placed in sterile, enzyme-free cryogenic vials, properly labeled, and transported in an ice box at 4°C. All tissue samples were stored in liquid nitrogen tanks until they were used for DNA extraction and sequencing. Clinical information was collected from patients through medical history and questionnaires.

### Immune cell distribution pattern evaluation of MGLSc prepuce samples

All MGLSc patients had their prepuce tissue collected for hematoxylin-eosin. Under double-blind conditions, three independent evaluators—an experienced pathologist and two urologists trained in lichen sclerosus (LS) pathological diagnostic protocols—conducted parallel assessments. Based on the degree and distribution patterns of inflammatory cell infiltration in tissue sections from MGLSc patients, the sections were classified into four types: “scattered,” “patchy or nodular,” “band-like,” and “lichenoid.” Scattered density showed lymphocytes sparsely infiltrating within the sclerosus dermis. Patchy and nodular densities showed lymphocytic infiltration in a patchy and nodular pattern within the sclerotic dermis. Band-like density showed lymphocytes exhibiting the contiguous band infiltration beneath the dermis sclerosus. Lichenoid density showed lymphocytes present in the basal layer of the epidermis and scattered within the epithelium (14, 15).

### Immunohistochemistry staining of bacterial markers on prepuce tissue

Deparaffinization and rehydration were performed on tissue sections using standard protocols. Heat-mediated antigen retrieval was conducted in 1 mmol/L Tris-EDTA buffer (pH 9.0) with 15 minutes of boiling followed by 15 minutes of thermal maintenance. Sections were rinsed three times with phosphate-buffered saline (PBS) (5 minutes each). After PBS removal, each section was treated with 100 µL (approximately one drop) of 5% bovine serum albumin blocking solution and incubated at room temperature for 20 minutes. Primary antibodies against lipoteichoic acid (LTA) (1:100; Hycult Biotech, HM2048) and lipopolysaccharide (LPS) (1:100, Hycult Biotech, HM6011) diluted in antibody solution were applied to each section (50 µL/section). Sections were incubated overnight at 4°C in a humidified chamber, followed by three PBS washes (5 minutes each). Subsequently, sections were incubated with secondary antibody (horseradish peroxidase-conjugated anti-mouse IgG) at room temperature for 30 minutes. Color development was initiated using DAB substrate (DAKO, BP0770) and immediately stopped upon observation of yellow granular or flaky precipitation. Residual DAB was thoroughly rinsed under running tap water. Counterstaining with hematoxylin for 30 seconds was followed by 5 minutes of tap water rinsing. Differentiation in hydrochloric acid-alcohol for 1 second preceded 10 minutes of tap water rinsing for bluing. After dehydration, sections were cleared in xylene with three 5 minute immersions prior to mounting (16) (see Fig. 4B and Fig. S3 for the representative staining images and negative control images).

### High-throughput 16S ribosomal RNA gene sequencing and downstream analysis

Total DNA was extracted from frozen prepuce samples using TGuide S96 Magnetic Soil /Stool DNA Kit (Tiangen Biotech [Beijing] Co., Ltd.) according to the manufacturer’s instructions. The specific primers employed were as follows: forward primer 27F (AGRGTTTGATYNTGGCTCAG) and reverse primer 1492R (TASGGHTACCTTGTTASGACTT). Following polymerase chain reaction (PCR) amplification, we conducted a quality assessment of the sequencing library and performed sequencing on the PacBio Sequel II platform (Beijing Biomarker Technologies Co., Ltd., Beijing, China).

The qualified sequences with more than 97% similarity thresholds were allocated to one operational taxonomic unit (OTU) using USEARCH (v.10.0). Taxonomy annotation of the OTUs/(amplicon sequence variants) was performed based on the Naive Bayes classifier in QIIME2 using the SILVA 138.1 database with a confidence threshold of 70%. Characterization of the alpha and beta diversities of microbial communities and their composition at the species level was conducted through QIIME2 software (v.2020.6). Linear discriminant analysis effect size (LEfSe) analysis was utilized to identify statistically significant biomarkers between the groups through the lefse package (v.1.1.2), with a threshold linear discriminant analysis (LDA) score greater than 4 set for biomarker selection. OTU sequences were compared against the Greengenes database to perform phenotype prediction analysis through the BugBase package (v.0.1.0) (17).

### Transcriptome sequencing and downstream analysis

Total RNA was extracted from frozen prepuce tissues using TRIzol Reagent (Life Technologies, California, USA). The mRNA libraries were prepared by poly-A selection, cDNA synthesis, fragmentation, adapter ligation, and PCR amplification through the Hieff NGS Ultima Dual-mode mRNA Library Prep Kit (Yeasen Biotechnology, Shanghai, China), then were sequenced on the Illumina NovaSeq 6000 platform. Raw reads underwent quality control with FastQC and trimming with Trimmomatic. HISAT2 (v.2.0.4) aligned reads to the *GRCh38* reference genome, and FeatureCounts quantified gene expression through the bioinformatics pipeline tool BMKCloud. DESeq2 (v.1.48.1) identified differentially expressed genes (DEGs) (adjusted *P* value of <0.05, |log2 fold change| >1). Differential gene functional annotation and enrichment analyses were performed using the Metascape (v.3.5.20250101). The top 15 enriched Gene Ontology (GO) pathways were categorized into different functional modules based on their GO functional descriptions.

Based on gene expression results, we conducted deconvolution analyses of immune cell infiltration through the single sample gene set enrichment analysis (ssGSEA) to calculate the enrichment score of 28 distinct immune cell types to compare cellular heterogeneity between the MGLSc and control groups. We used the “ssgsea” function from the GSVA package (v.2.2.0) for the analysis. Furthermore, to reveal the particular patterns of two groups, the immune cell infiltration scores in the two groups were evaluated using the ggpubr package (v.0.6.0) of R software (v.4.4.1), and statistical significance was assessed using the Wilcoxon test.

### Correlation analysis between microbiome and host gene expression

Statistical analyses were performed using R 4.4.1 to investigate two distinct correlative relationships: microbial richness with immune cell infiltration scores, and microbial relative abundance profiles with gene expression levels. Spearman’s rank correlation coefficient was employed, with genes exhibiting false discovery rate-adjusted *P* values of <0.05 considered statistically significant. dplyr (v.1.1.4) was used for data cleaning; reshape2 (v.1.4.4) was used for data formatting; and Hmisc (v.5.2-3) was used to obtain Spearman correlation and *P* values. Correlation analyses between microbial abundance and immune cell infiltration scores were systematically conducted across two dimensions: inter-group comparisons of MGLSc patients versus controls and intragroup evaluations within each cohort (18).

Then, we analyzed the correlation between microbial abundance and immune cell abundance within each group to observe whether microbe-host interactions vary across different populations (18). We also utilized the “cor.test” function of stats package (v.4.4.1) to identify genes significantly correlated with differential microbes in MGLSc and controls, respectively (*P* < 0.05). Genes belonging to the “immune activation,” “inflammatory response,” and “innate immune and pathogen response” modules were then subjected to correlation analyses with differential microbes in both MGLSc and controls.

### Correlation analysis between microbiome and clinical data of the MGLSc cohort

Given that MGLSc is closely associated with chronic inflammatory diseases (19), correlation analysis was performed between microbes and risk factors related to MGLSc, including age, body mass index (BMI), blood pressure, diabetes, hyperlipidemia, lymphocyte distribution patterns, stenosis grade, and stenosis score in the MGLSc urethral stricture group (19). The evaluation of the stenosis grade and the stenosis score of MGLSc was conducted based on the Trauma and Urologic Reconstruction Network of Surgeons (TURNS) classification system for urethral strictures (Supplemental materials) (20). Based on the types of clinical data, the association between binary variables and microbes was represented by calculating the point-biserial correlation coefficient. For continuous clinical variables, the association with microbes was represented by calculating the Spearman correlation coefficient. Each clinical data point was analyzed separately for its association with the microbes. All analyses were based on data collected from the enrolled participants through medical history and the questionnaires.

## RESULTS

### Participant information

The study cohort comprised 40 MGLSc patients and 30 redundant prepuce controls. From the MGLSc group, 13 patients were excluded: 4 with prior circumcision history, 4 with recent antibiotic use (within 3 months), 3 with active urinary tract infections confirmed by urine culture, and 2 failing 16S rRNA sequencing quality controls. The control group excluded 13 individuals aged below 18 years through stringent age stratification. The final study cohort included 27 MGLSc patients and 17 controls with redundant prepuce. All participants underwent full-length 16S rRNA gene sequencing, with 23 MGLSc patients and 11 controls having sufficient residual tissue for transcriptome sequencing.

Demographic data showed a mean age of 52.2 years (24–77) in MGLSc patients versus 28.5 years (18–48) in controls. The median BMI was 24.6 kg/m^2^ (17.3–42.3) and 23.6 kg/m^2^ (19.2–30.5), with no significant differences observed between the two groups (*P* = 0.116). Comorbidities were exclusively observed in the MGLSc group: three cases of hyperglycemia (fasting glucose ≥7.0 mmol/L) and five cases of hyperlipidemia (low-density lipoprotein ≥3.4 mmol/L), whereas none were detected in controls. Urethral stricture severity in MGLSc patients was classified using the TURNS Length, Segment, Etiology Anterior Urethra Classification System (21, 22). Surgical interventions included urethroplasty in 21 patients (77.8%) and perineostomy in 6 patients (22.2%). Control subjects underwent routine circumcision without perioperative complications (Table 1).

**TABLE 1:** Characteristics of the MGLSc group and the control group included in the cohort^*c*^

Patient characteristics	Group		*P* value
	MGLSc-US (*n* = 27)	Healthy control (*n* = 17)	
Age (year), mean ± SD (range)	52.2 ± 12.3 (24–77)	28.5 ± 7.8 (18–48)	<0.001^*a*^
BMI (kg/m^2^), median (range)	24.6 (17.3–42.3)	23.6 (19.2–30.5)	0.116^*a*^
Diabetes mellitus, *n*	3	0	0.272^*b*^
Hyperlipidemia, *n*	5	0	0.139^*b*^
Stricture grade, median (range)	2 (1–3)	N/A	N/A
Structure score, median (range)	8 (6–11)	N/A	N/A
Surgery, *n* (%)			
Urethroplasty	21 (78)	N/A	N/A
Perineostomy	6 (22)	N/A	N/A
Circumstance	N/A	17 (100)	N/A

^*a*^ *P* value: Welch’s *t*-test.

^*b*^ *P* value: Fisher’s exact test.

^*c*^ BMI, body mass index; N/A, not available; MGLSc-US, male genital lichen sclerosus-induced urethral stricture.

### Composition and diversity of MGLSc prepuce microbiota and normal prepuce microbiota

**Fig 1:**
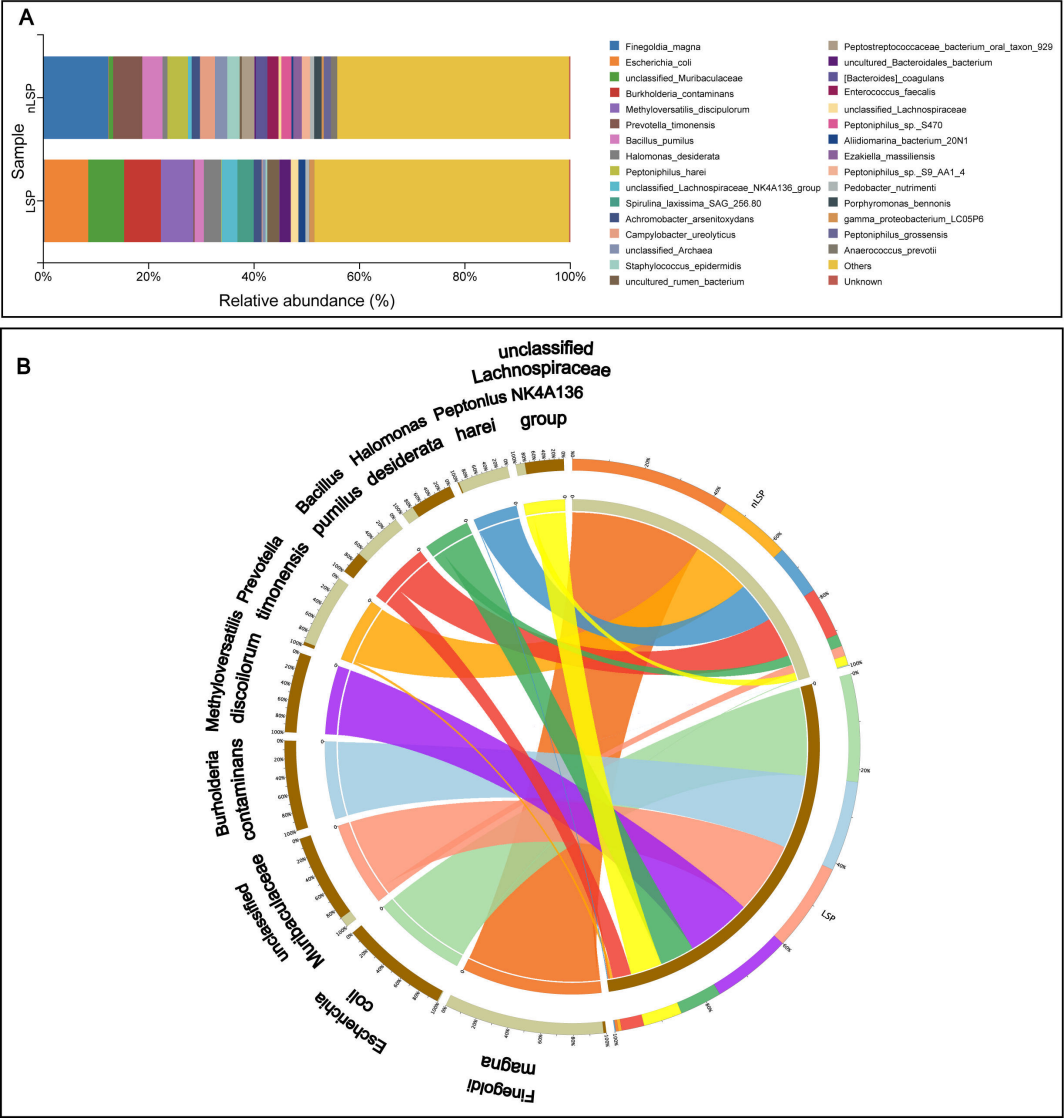
Microbiome composition in the prepuce of the MGLSc and control groups. (**A**) Bar chart of species composition at the species level. The top 30 most abundant microbes based on average relative abundance are shown for the lichen sclerosus prepuce lesion (LSP) (MGLSc) and non-lichen sclerosus prepuce lesion (nLSP) (Control) groups. (**B**) Circos plot of community composition. The microbial community structure differs between the LSP (MGLSc) and the nLSP (Control) groups. Microbes that are highly ranked in abundance within one group show a significantly reduced abundance in the other group. Different patient groups and microbes were labeled using corresponding colors.

**Fig 2:**
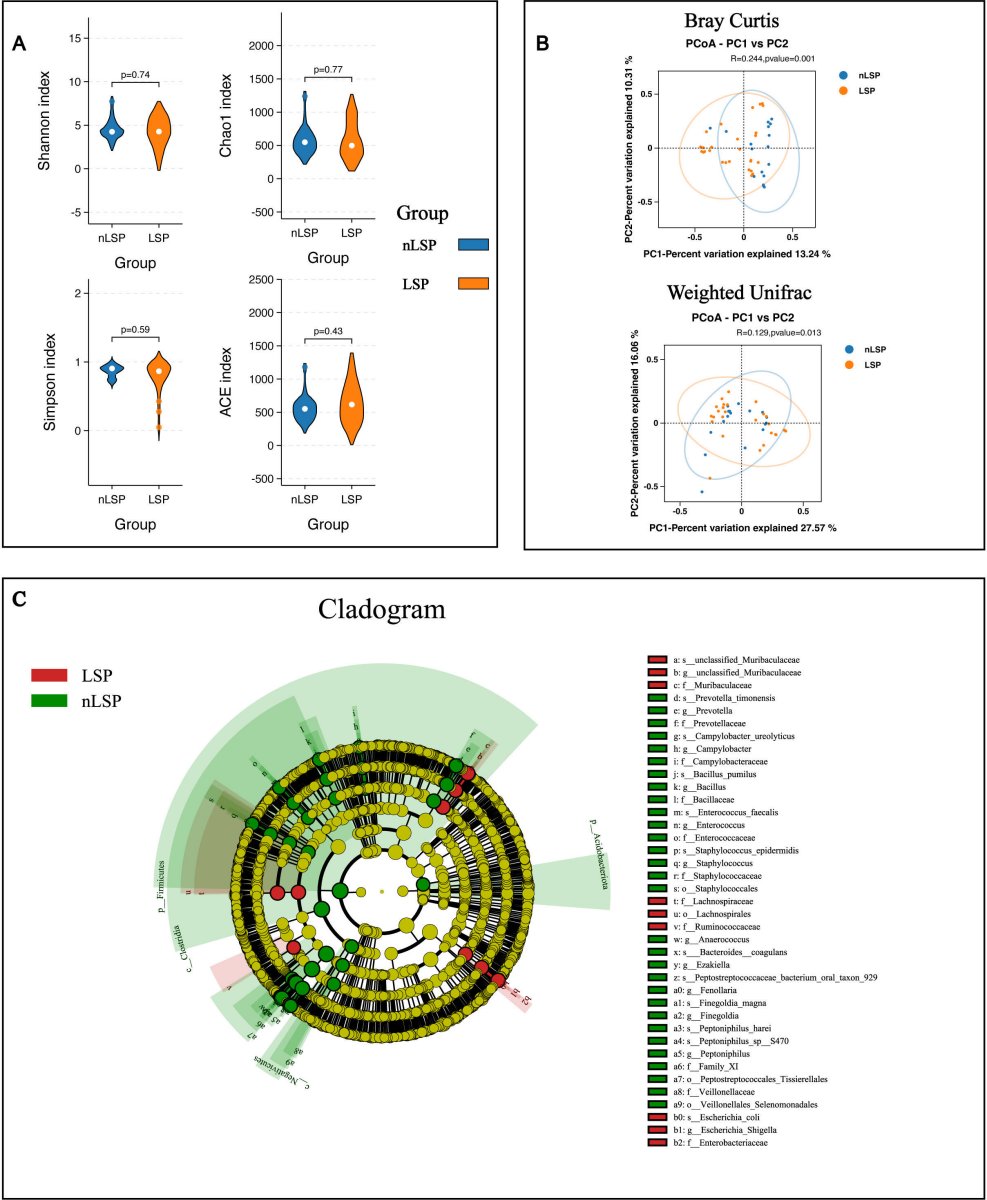
Differences in microbial composition between the MGLSc and control groups. (**A**) Alpha diversity indices. A comparison of microbial composition at the species level between the LSP (MGLSc) and nLSP (control) groups, showing the Shannon index (*P* = 0.74), Chao 1 index (*P* = 0.77), Simpson index (*P* = 0.59), and ACE index (*P* = 0.43). (**B**) Principal coordinate analysis (PCoA). The microbial composition at the species level differs significantly between the LSP (MGLSc) and nLSP (control) groups, as shown by the Bray-Curtis algorithm (*R* = 0.244, *P* = 0.001) and the weighted UniFrac algorithm (*R* = 0.129, *P* = 0.013). (**C**) LEfSe analysis for comparing microbial variations in LSP and nLSP from phylum to species levels. The LEfSe cladogram represents differentially abundant taxa (*P* < 0.05); LDA scores are calculated based on the LEfSe analysis of differentially abundant taxa among groups, and only taxa with LDA scores greater than 4 are presented.

According to the relative abundance analysis of taxa at the species level in samples, MGLSc and normal prepuce had unique tissue microbiome compositions. The top nine most abundant microbes in each group’s microbiota were entirely different. These microbes formed the main part of each group’s microbiota, with the top nine microbes accounting for 37.77% of the microbiota in the control group and 41.99% in the MGLSc group. Microbes with high abundance in the control group showed extremely low abundance in the MGLSc group, while those with high abundance in the MGLSc group were extremely low in abundance in the control group. (Fig. 1A and B; Fig. S1). In addition, alpha diversity indices including Simpson (*P* = 0.079), Chao 1 (*P* = 0.14), Shannon (*P* = 0.2), and abundance-based coverage-estimator (ACE) (*P* = 0.26) did not show significant differences between the MGLSc and control groups (Fig. 2A). Notably, beta diversity analysis using Bray-Curtis (*R* = 0.244, *P* = 0.001) and weighted UniFrac (*R* = 0.128, *P* = 0.008)-based principal coordinate analyses revealed significant differences between the MGLSc and normal prepuce samples (Fig. 2B). These results indicated that the tissue microbiome dysbiosis significantly existed in MGLSc prepuce lesions compared to normal prepuce.

Furthermore, the LEfSe analysis identified species-level dominant and differential microbes in both normal prepuce and MGLSc prepuce. In the MGLSc group, the abundance of unclassified Muribaculaceae and *Escherichia coli* significantly increased (*P* < 0.05, ∣LDA∣ >4) (Fig. S2). In contrast, the abundance of *Finegoldia magna*, *Prevotella timonensis*, *Campylobacter ureolyticus*, *Bacillus pumilus*, *Enterococcus faecalis*, *Staphylococcus epidermidis*, *Bacteroides coagulans*, Peptostreptococcaceae bacterium oral taxon 929, *Peptoniphilus harei*, and *Peptoniphilus* sp. S470 significantly decreased in the MGLSc group (*P* < 0.05, ∣LDA∣ >4) (Fig. 2C; Fig. S2).

### BugBase phenotype prediction analysis and microbiota distribution patterns of microbes in MGLSc prepuce and normal prepuce

**Fig 3:**
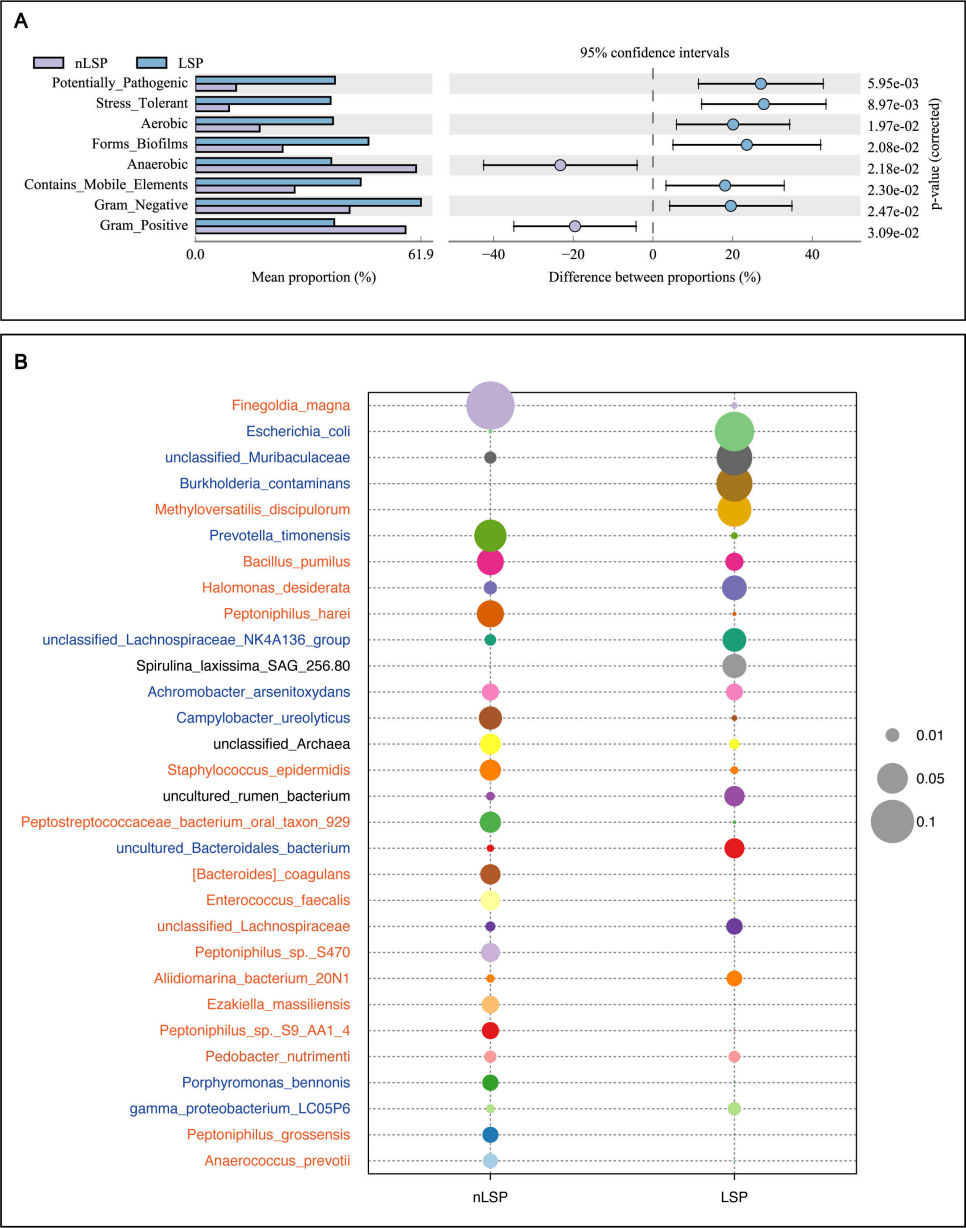
Microbial phenotypic characteristics in the MGLSc prepuce. (**A**) BugBase phenotype prediction analysis. The left side shows the abundance ratio of different functions in two samples or groups of samples; the middle displays the difference in functional abundance within a 95% confidence interval; and the value on the far right indicates the *P* value. (**B**) Bubble plot of the average top 30 most abundant species at the species level. Bacteria classified as gram positive are labeled in orange, while those classified as gram negative are labeled in blue. Bacteria that cannot be specifically classified as gram positive or gram negative are indicated in black font. The size of the bubbles represents the proportion of each species within the group.

In the microbial functional prediction analysis, the microbial communities colonizing the prepuce tissue of MGLSc patients showed a significantly lower proportion of gram-positive bacteria and exhibited a greater potential pathogenic capability. The microbes in the MGLSc group exhibit greater biofilm formation ability, pathogenicity, and environmental tolerance (Fig. 3A). In the microbiomes of the two groups, among the top 30 most abundant microbes on average, gram-positive bacteria accounted for approximately 39.4% of the microbiome in the control group and about 15.4% in the MGLSc group. In contrast, gram-negative bacteria accounted for about 13.6% in the control group and approximately 30.1% in the MGLSc group (Fig. 3B).

**Fig 4:**
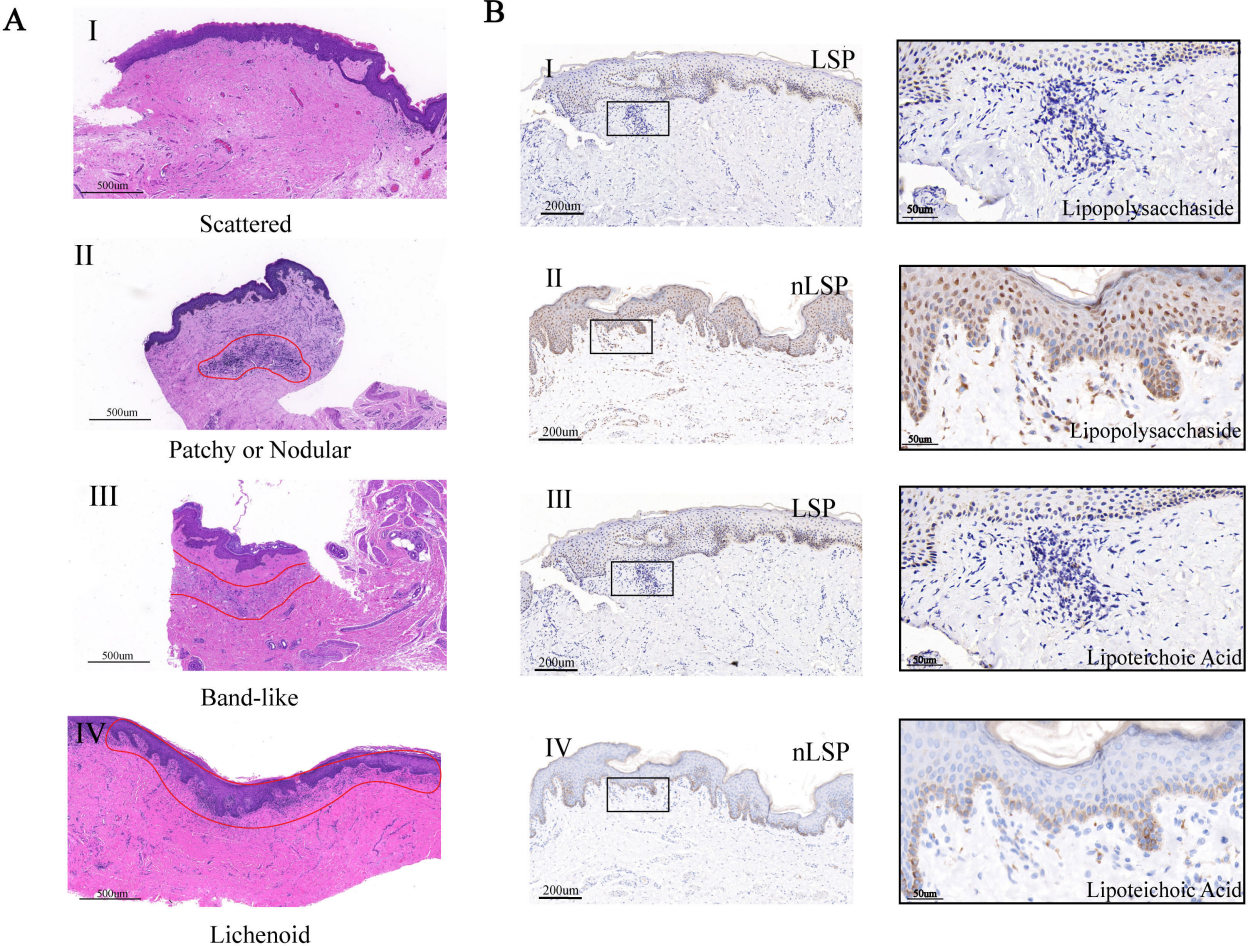
Lymphocyte infiltration pattern of MGLSc prepuce and distribution of gram-positive and gram-negative bacteria in MGLSc and normal prepuce. (**A**) Lymphocyte distribution pattern of MGLSc prepuce (×10). (AI–IV) The histopathological types of MGLSc prepuce were primarily classified into four categories based on the distribution patterns of inflammatory cells: scattered, patchy or nodular, band-like, and lichenoid. (**B**) Immunohistochemical staining of bacterial markers in foreskin sections. Middle (×20), right (×63). (BI) Distribution of gram-negative bacteria in MGLSc lesional foreskin tissue. (BII) Distribution of gram-negative bacteria in control group foreskin tissue. (BIII) Distribution of gram-positive bacteria in MGLSc lesional foreskin tissue. (BIV) Distribution of gram-positive bacteria in control group foreskin tissue. Brown indicates bacterial marker-positive areas, and blue indicates nuclei.

Based on the results of microbial functional prediction analysis, to further explore the spatial distribution pattern of microbes in MGLSc and normal prepuce, we performed MGLSc prepuce histological examination and immunohistochemistry staining of gram-positive and gram-negative bacteria in the two groups. According to the distribution patterns of inflammatory cells in the lesional prepuce, the samples were classified into four categories: scattered (*n* = 5, 18.52%), patchy or nodular (*n* = 11, 40.74%), band-like (*n* = 5, 18.52%), and lichenoid (*n* = 5, 18.52%). Patchy or nodular was the main distribution pattern in MGLSc prepuce (Fig. 4A). Inter-rater reliability analysis demonstrated substantial agreement with an intraclass correlation coefficient of 0.742 (95% confidence interval [CI] 0.703–0.907) and a Fleiss kappa coefficient of 0.653 (95% CI 0.494–0.812). Complete concordance was achieved in 60.7% of cases, with discrepancies (3.6%) resolved through consensus panel review.

In addition, although all enrolled participants were in the non-acute infection phase, we still could find that gram-negative and gram-positive bacteria were distributed in both groups’ prepuce tissue (Fig. 4B; Fig. S5). In normal prepuce, gram-negative and gram-positive bacteria were distributed in the epidermal layer (Fig. 4B; Fig. S5). Notably, in the dermis of MGLSc prepuce lesions, microbial staining was observed within specific inflammatory cell infiltration zones. Normal prepuce did not have inflammatory cell infiltration zones (Fig. 4B).

### Transcriptome and immune infiltration analysis in MGLSc prepuce and normal prepuce

**Fig 5:**
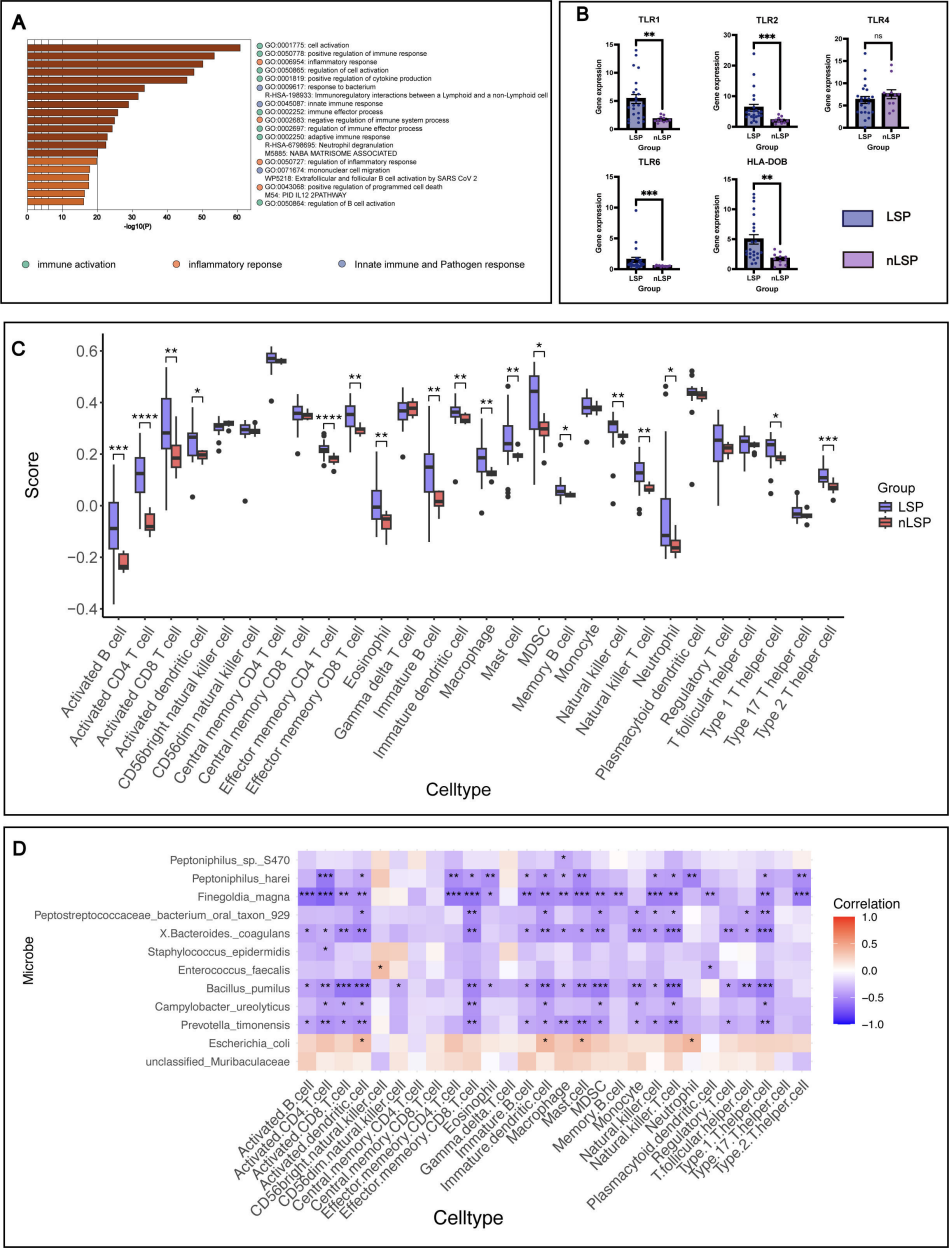
Transcriptome and immune infiltration features of the nLSP and LSP groups. (**A**) Bar chart of functional annotation of differentially expressed genes. The top 15 GO functional annotations categorize the differentially expressed genes into three major modules: innate activation, inflammatory response, and innate immune and pathogen response. (**B**) Bar chart of gene expression related to microbial recognition. The bars in the bar chart represent the mean of the data, and the error bars above or below the bars are based on the standard error of the mean. (**C**) Immune infiltration analysis results showing differences in immune cell abundance between the LSP and nLSP groups. (**D**) Heatmap of the correlation between microbial abundance and immune cell abundance including all patients from the MGLSc and control groups. *P < 0.05, **P < 0.01, ***P < 0.001, and ****P < 0.0001.

Among 23 MGLSc patients and 11 control patients, a total of 1,243 differentially expressed genes were identified, with 1,139 upregulated genes and 104 downregulated genes (Fig. S3). Functional annotation and enrichment of DEGs in MGLSc patients revealed that the top 20 pathways primarily focus on immune activation and regulation. The top 15 enriched GO pathways were categorized into three functional modules: “immune activation” (“GO:0001775,” “GO: 0050778,” etc.), “inflammatory response” (“GO:0006954,” “GO:0002683,” etc.), and “innate immune and pathogen response” (“GO:0009617,” “GO:0045087,” etc.) (Fig. 5A). In the MGLSc population, the expression levels of *TLR1*, *TLR2*, and *TLR6*, which are associated with pathogen recognition, were significantly elevated (Fig. 5B). Additionally, the expression levels of genes from the matrix metalloproteinase (MMP) family, which are involved in extracellular matrix degradation and inflammation, also showed a significant upregulation, including *MMP7*, *MMP9*, *MMP11*, *MMP12*, and *MMP25* (Fig. S4). The characteristics of immune dysregulation in MGLSc were explored through ssGSEA. The immune cell infiltration analysis showed a significant increase in innate (e.g., macrophages and dendritic cells) and adaptive (e.g., activated CD4, CD8, type 1 T cells, and type 2 T cells) immune cells in the MGLSc group compared to the control group, indicating elevated immune activity across various immune processes (Fig. 5B and C).

### Transcriptome and microbiome integration analysis in MGLSc prepuce and normal prepuce

Dominant bacteria in normal prepuce, such as *Finegoldia magna* and *Prevotella timonensis*, showed significantly negative correlation with the immune cell infiltration score. The dominant bacteria in MGLSc prepuce, such as unclassified Muribaculaceae and *Escherichia coli*, showed positive correlations with immune cells. Notably, *Escherichia coli* was significantly positively correlated with neutrophils, mast cells, immature dendritic cells, and activated dendritic cells. Among all bacteria, *Finegoldia magna* showed the strongest correlation with immune cells activated in the MGLSc prepuce, except for neutrophils (Fig. 5D).

**Fig 6:**
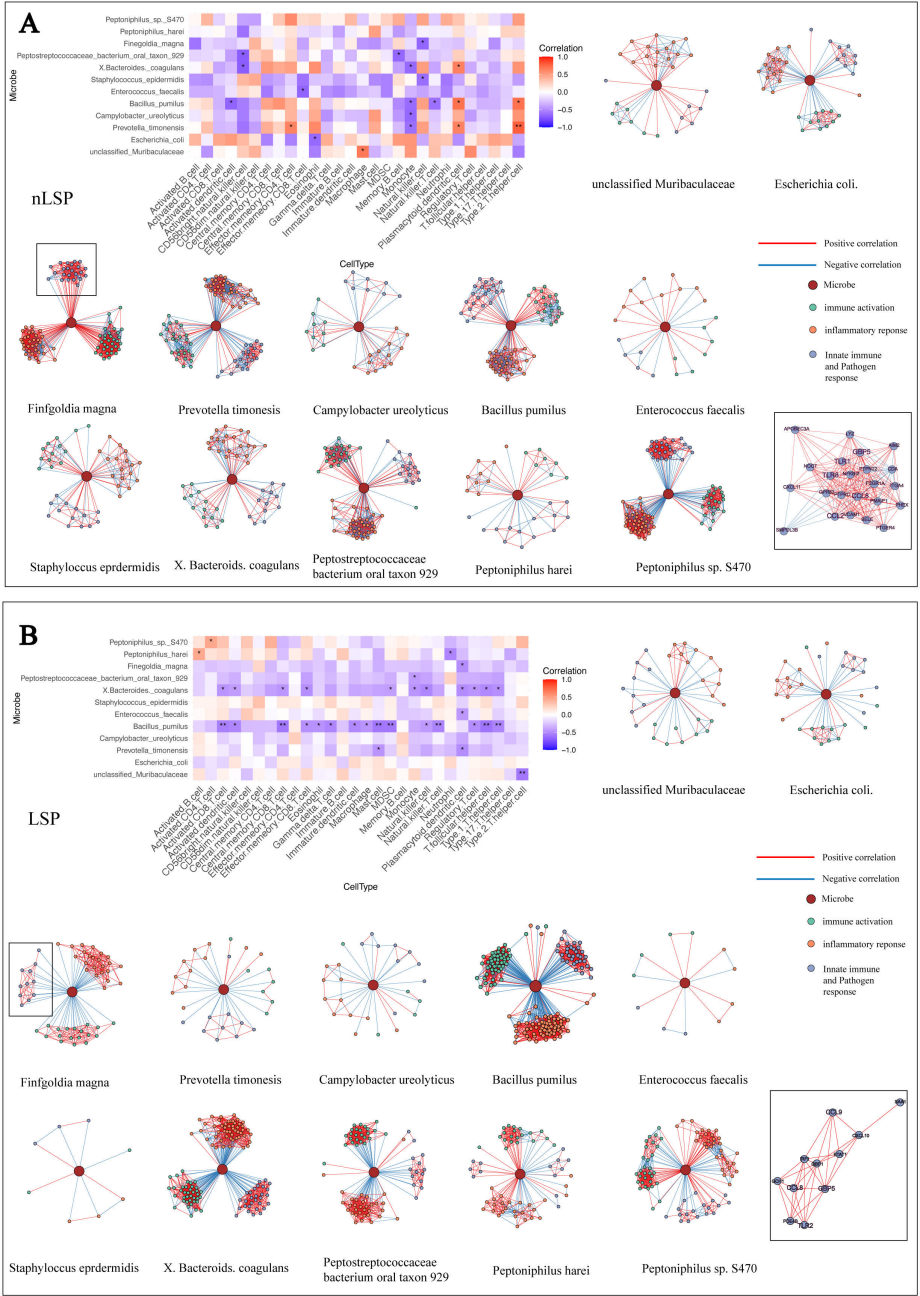
Correlation between microbial abundance, immune cell abundance, and gene expression in the nLSP and LSP groups. (**A**) In the nLSP group, the heatmap of the correlation between microbial and immune cell abundance, along with a network diagram showing the correlation between microbes and genes within the three modules: innate activation, inflammatory response, and innate immune and pathogen response. (**B**) In the LSP group, the heatmap of the correlation between microbial and immune cell abundance, and a network diagram depicting the correlation between microbes and genes within the three modules: innate activation, inflammatory response, and innate immune and pathogen response. *P < 0.05 and **P < 0.01. Circles in different colors represent genes from different modules, and the circle at the center of the network diagram represents the microorganism. Red lines indicate positive correlations between genes and the microorganism and between the genes, while blue lines indicate negative correlations between genes and the microorganism and between the genes.

In the control group, dominant bacteria in the control cohort (like *Finegoldia magna*) were positively correlated with plasmacytoid dendritic cells and Th2 cell infiltration. These microbes were negatively correlated with monocyte, natural killer (NK) cell, and natural killer T (NKT) cell infiltrations. In addition, these bacteria significantly positively correlated with pathways involved in immune activation, inflammatory response, and innate immune and pathogen response. Notably, three functional module genes were positively correlated with these microbial abundances. These microbes positively correlated with *GBP5* and CCL8, TLR1, TLR2, and TLR6 in the innate immune and pathogen response module (Fig. 6A).

Conversely, these microbes had low abundance in MGLSc prepuce and were not only negatively correlated with monocyte, NK cell, and NKT cell infiltrations but also negatively correlated with Th1 cells, activated CD8 T cells, central memory CD8 T cells, effector memory CD8 T cells, and so forth. Moreover, these microbes exhibited different trends in their correlation with three pathway modules and genes compared to those in normal prepuce, which suggested that most microbes showed a decrease in abundance as the expression of these genes increased (Fig. 6A and B).

### Differential microbe abundance and clinical phenotype correlation analysis in MGLSc-US patients

**Fig 7:**
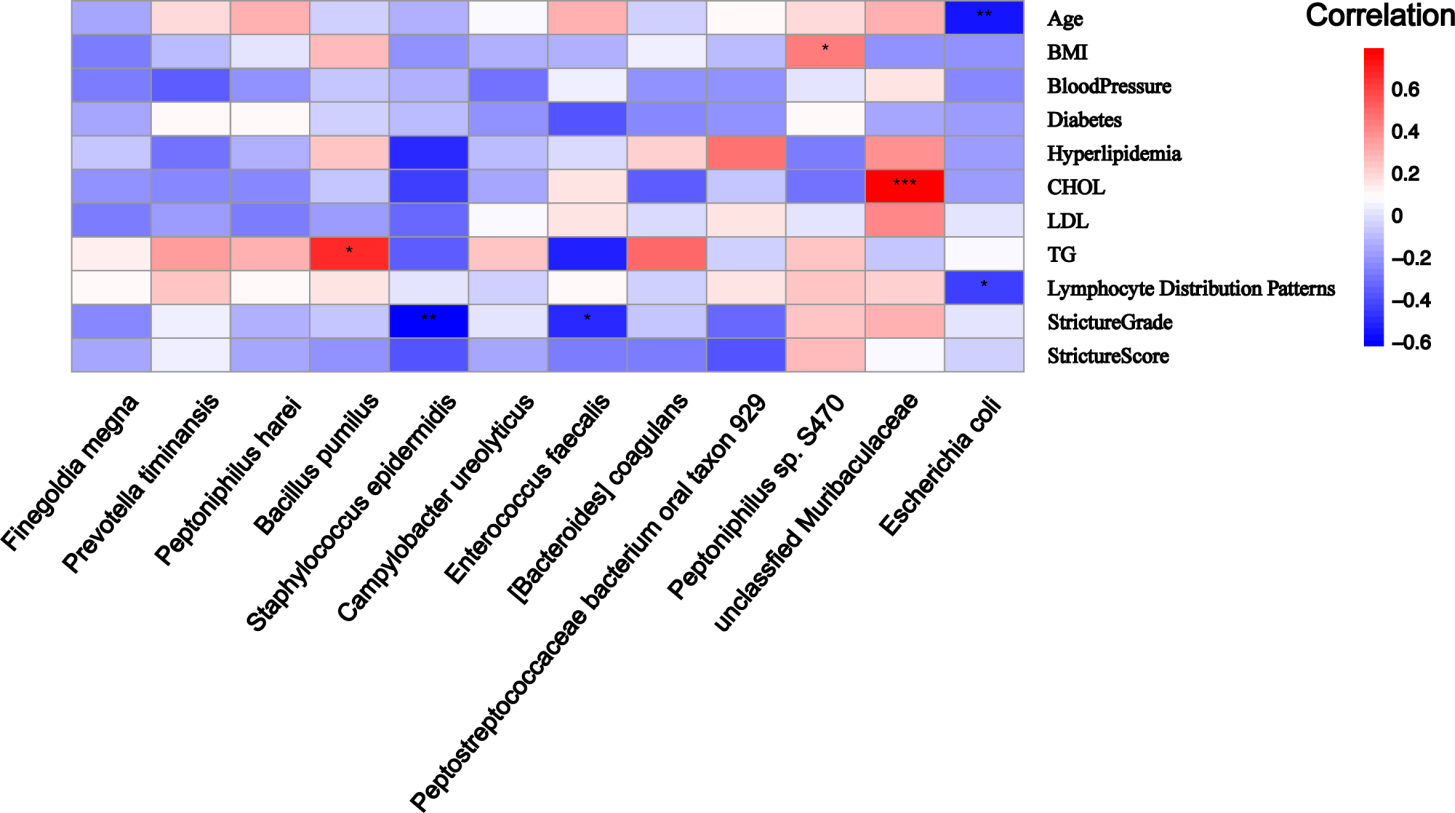
Heatmap of the correlation between microbial abundance and clinical data of patients in the MGLSc group. Red denotes a positive correlation, and blue denotes a negative correlation. *P < 0.05, **P < 0.01, ***P < 0.001.

*Escherichia coli* was negatively correlated with the patient’s age (*R* = −0.541, *P* = 0.004) and the lymphocyte infiltration pattern (*R* = −0.441, *P* = 0.024). The higher the abundance of *Escherichia coli* in the prepuce tissue of MGLSc patients, the sparser the distribution of lymphocytes in the tissue. The unclassified Muribaculaceae was positively correlated with total cholesterol (CHOL) levels (*R* = 0.793, *P* = 0.0007). In the low abundance of distinct bacteria in the MGLSc prepuce, the abundance of *Peptoniphilus* sp. S470 was positively correlated with BMI (*R* = 0.433, *P* = 0.024). The abundance of *Bacillus pumilus* was positively correlated with triglyceride levels (*R* = 0.652, *P* = 0.012). *Enterococcus faecalis* and *Staphylococcus epidermidis* were negatively correlated with stenosis grading (Fig. 7). The fewer *Enterococcus faecalis* and *Staphylococcus epidermidis* present, the longer the stricture segments.

## DISCUSSION

Previous studies have demonstrated microbial dysbiosis on urine and the prepuce swab in MGLSc patients. However, the nature of the interactions between these microbes and the host remains poorly understood. In this study, we first described the unique tissue microbiome composition and provided the preliminary clues of host-microbe interactions within the MGLSc prepuce tissue.

In microbiome diversity, we found alpha diversities did not show a significant difference, but beta diversities showed significant difference. These results were similar to the previous research in urine and balanopreputial sac swab (3). Additionally, in MGLSc prepuce lesions, the abundance of normal prepuce dominant microbes, such as *Finegoldia magna*, *Prevotella timonensis*, and *Staphylococcus epidermidis*, was decreased (Fig. 2C). Similarly, in the balanopreputial swab, *Finegoldia magna* was also found to have a higher abundance in the control group compared to the MGLSc group (23). *Finegoldia magna* was reported as a common microbe on the skin but could cause infections when the skin was damaged or the host’s immunity was compromised (24). *Prevotella timonensis*, though commonly detected in healthy skin, could cause bacterial vaginosis under certain conditions (25). *Staphylococcus epidermidis* was considered related to skin barrier homeostasis. Notably, the high-abundance distinct microbes were unclassified Muribaculaceae and *Escherichia coli*. *Escherichia coli* was a common gut microbe and commonly associated with urinary tract infection (23). Muribaculaceae was also a common gut microbe and involved in producing short-chain fatty acids (SCFAs) (26). Interestingly, these microbes were closely associated with the urinary tract (27, 28), and our cohort of MGLSc patients had a history of urethral stricture and genital structural remodeling, increasing the likelihood of these microbes colonizing the prepuce.

The upregulated DEGs were predominantly associated with immune activation, inflammatory response, and innate immune and pathogen response. Notably, the results of ssGSEA showed a significant increase in activated B cells and Th2 cells in the MGLSc group. The significant difference in effector memory CD4 T cells between the two groups indicates persistent antigen exposure in the MGLSc group, leading to long-term retention and immune surveillance of memory T cells (29). Corresponding to these results, in the MGLSc group, genes in the Toll-like receptor (TLR) family that primarily recognized gram-positive bacterial markers, such as TLR1, TLR2, and TLR6 (30), were highly expressed in the lesional tissues, while the expression of TLR4, which primarily recognized gram-negative bacterial markers (31), showed no significant difference between the two groups. The observed divergence between 16S rDNA sequencing and transcriptomic data may be attributed to residual ligand sensing. Following bacterial death mediated by host immunity or microbial competition, persistent cell wall components act as residual ligands, sustaining *TLR-2/6* activation (32, 33). Although *TLR-4* did not show the significant difference between two groups, gram negativity may still be involved in inflammatory response, as *Escherichia coli*’s Braun lipoprotein has been shown to activate *TLR-2* via cross-reactivity mechanisms (34).

What is more important is that differential dominant microbial taxa (like *Finegoldia magna*) of the control group in MGLSc patients’ prepuce tissues demonstrated negative correlation with immune cell infiltration scores. *Finegoldia magna* in MGLSc patients’ prepuce tissues also exhibited a negative correlation with DEGs (*GBP5*, *CCL8*, etc.). *GBP5* and CCL8 were associated with the activation of the nuclear factor kappa-light-chain-enhancer of activated B cells (NF-κB) signaling pathway (35, 36). These results suggested that *TLR2/6* and NF-κB-mediated host immune activation may contribute to the lytic clearance of gram-positive bacteria in MGLSc lesions. Additionally, we also found Muribaculaceae was significantly positively correlated with CHOL levels. Unclassified Muribaculaceae was reported to produce SCFA, and the excessive SCFA on the skin could promote the expression of the *HIF1A* gene, potentially implicating dysbiosis in the initiation of cutaneous inflammation (37).

Notably, MMP3, MMP7, MMP9, and MMP25 were highly expressed in the lesional tissues of the MGLSc group, suggesting that the skin barrier may be compromised (38). The high expression of these genes could cause compromised skin barrier function through damaged basement membrane. This disruption could lead to the release of pro-inflammatory cytokines, amplifying the inflammatory response in MGLSc tissues and forming the inflammatory loop. Taken together, our findings support a hypothetical model in which epithelial barrier disruption in MGLSc facilitates microbial recognition through *TLR2/6*, leading to NF-κB-mediated inflammatory signaling and the lytic clearance of commensal gram-positive bacteria (39). Gram-negative bacteria may further enhance this response via *TLR2* cross-reactivity or through metabolite-associated immunomodulation. These host-microbiota interactions may establish a self-perpetuating inflammatory loop that contributes to sustained immune activation and epithelial remodeling in MGLSc.

Although our study presents exploratory findings on local host-microbiota interactions within MGLSc preputial lesions, it has several limitations that should be acknowledged. First, based on post hoc power analysis, the current sample size yields an estimated statistical power of 65%, which is below the ideal threshold for detecting the observed effect size. This limitation is largely due to the low incidence of MGLSc (0.1%–0.3%) (40), insidious onset of MGLSc (41), and our strict inclusion criteria. Nonetheless, our sample size is comparable to previous microbiome studies in LS, but larger cohorts will be required for future validation. Second, the cross-sectional design limits causal inference, and future studies incorporating cellular and animal models are needed to experimentally validate the proposed mechanisms. Third, this study was a single-center study, which may introduce some geographic and demographic bias. Confounding variables such as age, preputial hygiene habit, comorbidity, MGLSc duration, and treatment history were not fully controlled. Fourth, this study lacked microbial functional analysis. Metagenomic and metabolomic approaches should be employed to further explore microbial metabolism or host-microbe interaction at the mechanistic level. Finally, the immunohistochemistry results for LTA and LPS were qualitative, and more quantitative or high-resolution microbial characterization methods should be considered in future work. Future research should adopt multi-center, longitudinal cohort designs, with careful adjustment for potential confounders, integration of multi-omics data (metagenomics and metabolomics), and validation through functional assays in cellular and animal models to establish causality and mechanistic understanding.

### Conclusion

This study is the first to demonstrate a distinct microbial signature characterized by reduced gram-positive bacterial abundance in the preputial lesions of MGLSc patients. The multi-omics correlation analyses showed that this microbial dysbiosis might be linked to the activation of pathogen recognition pathways, particularly TLR2/6 and NF-κB, suggesting that localized immune pressure could contribute to the lytic clearance of commensal gram-positive taxa. As an exploratory analysis, our study proposes a potential model of host-microbiota interaction in MGLSc and identifies several microbial taxa associated with clinical features such as age, BMI, and cholesterol levels. These findings offer a conceptual foundation for future studies aiming to identify potential microbial biomarkers for diagnosis and stratified management of MGLSc.

## Data Availability

The raw RNA sequence data and 16S rDNA data reported in this article have been deposited in the Genome Sequence Archive (GSA-Human: HRA009122) and are publicly accessible.

## SUPPLEMENTAL MATERIAL

Supplemental materials and methods, and Fig. S1 to S5. spectrum.00074-25-s0001.docx Supplemental materials and methods: Sample size rationale and post-hoc power analysis. Fig. S1: Microbial composition. Fig. S2: Differential microorganisms. Fig. S3: Volcano plot of differentially expressed genes. Fig. S4: Differences in MMP genes between groups. Fig. S5: Supplementary results of immunohistochemical staining.

Table S1 spectrum.00074-25-s0002.docx STROBE Statement-checklist of items that should be included in reports of observational studies.

ASM does not own the copyrights to Supplemental Material that may be linked to, or accessed through, an article. The authors have granted ASM a non-exclusive, world-wide license to publish the Supplemental Material files. Please contact the corresponding author directly for reuse.
