# Supplementary material for: Multi-omics analysis reveals the host-microbe interactions on the dysbiosis of tissue microbiota in male genital lichen sclerosus-induced urethral strictures
Source: Microbiol Spectr. 2025 Aug 18;13(10):e00074-25. doi: 10.1128/spectrum.00074-25 (PMC12502673; doi:10.1128/spectrum.00074-25)
Supplement: Supplemental materials and methods, and Fig. S1 to S5. — Supplemental materials and methods: Sample size rationale and post-hoc power analysis. Fig. S1: Microbial composition. Fig. S2: Differential microorganisms. Fig. S3: Volcano plot of differentially expressed genes. Fig. S4: Differences in MMP genes between groups. Fig. S5: Supplementary results of immunohistochemical staining. [file spectrum.00074-25-s0001.docx]

**Supplementary Materials**

**Materials and Methods**

**Sample size rationale and post-hoc power analysis**

To retrospectively evaluate the adequacy of the sample size, a post-hoc power analysis was performed based on *Finegoldia magna*, one of the differentially abundant taxa identified by LEfSe. Using the standard formula for comparing two independent proportions:

$$n=\frac{\left( Z_{1-\frac{\alpha}{2}}+Z_{1-\beta} \right)^{2}\times\left( P_{1}\left( 1-P_{1} \right)+P_{2}\left( 1-P_{2} \right) \right)}{\left( P_{1}-P_{2} \right)^{2}}$$

$P_{1}$ = the proportion in the first group; $P_{2}$= the proportion in the second group; $Z_{1-\frac{\alpha}{2}}$= 1.96 (α=0.05), $Z_{1-\beta}$=0.84 (β=0.20); $P_{1}$－$P_{2}$= Effect Size (difference in proportions).

The theoretical sample size was calculated as 57 subjects per group using standard parameters (α = 0.05, power = 80%). However, given the low incidence rate of the target disease (0.1%-0.3% in general population with even lower rates in males) as reported in classical LS studies, eligible cases meeting inclusion criteria were limited. Based on strict inclusion and exclusion criteria, practical enrollment reached 27 cases in the disease group and 17 controls. The study demonstrated 65% statistical power under the observed effect size (12.6%) with current sample size.

**Evaluation methods of stricture grade and stricture score.**

MGLSc-US (Male genital lichen sclerosis urethral stricture) patients are classified into three grades based on urethral stricture length: Grade 1 for strictures ≤2 cm, Grade 2 for strictures >2 cm and ≤7 cm, and Grade 3 for strictures >7 cm. Additionally, a scoring system is used to evaluate the severity of the stricture, considering three factors: length, segment, and etiology. For length, strictures ≤2 cm receive 1 point, those >2 cm and ≤7 cm receive 2 points, and strictures >7 cm are assigned 3 points. The segment of involvement is scored as follows: bulbar urethral without distal bulbar involvement receives 1 point, penile urethral with or without meatal and/or fossa navicularis involvement receives 2 points, bulbar urethral with distal bulbar involvement is given 3 points, and panurethral involvement (bulbar and penile urethral segments without meatal or fossa navicularis involvement) receives 4 points. Lastly, the etiology is evaluated, with external traumatic causes receiving 1 point, idiopathic/unknown causes, internal iatrogenic trauma, recurrent strictures after prior urethroplasty, infections/inflammation, or hypospadias receive 2 points, and radiation or lichen sclerosis receiving 3 points.

**Supplementary Figures**

**
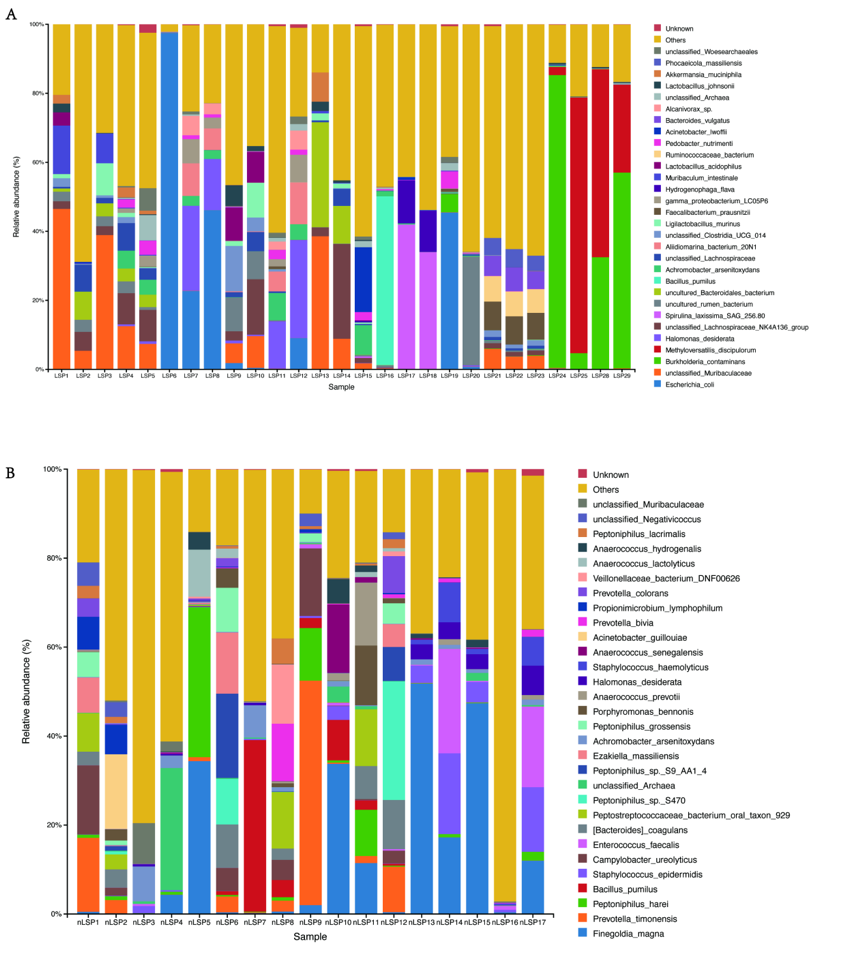
**

**Fig. S1** **(A)** Microbial species abundance bar chart in the prepuce of the MGLSc Group**. (B)** Microbial abundance bar chart in the prepuce of the control group**.**

**
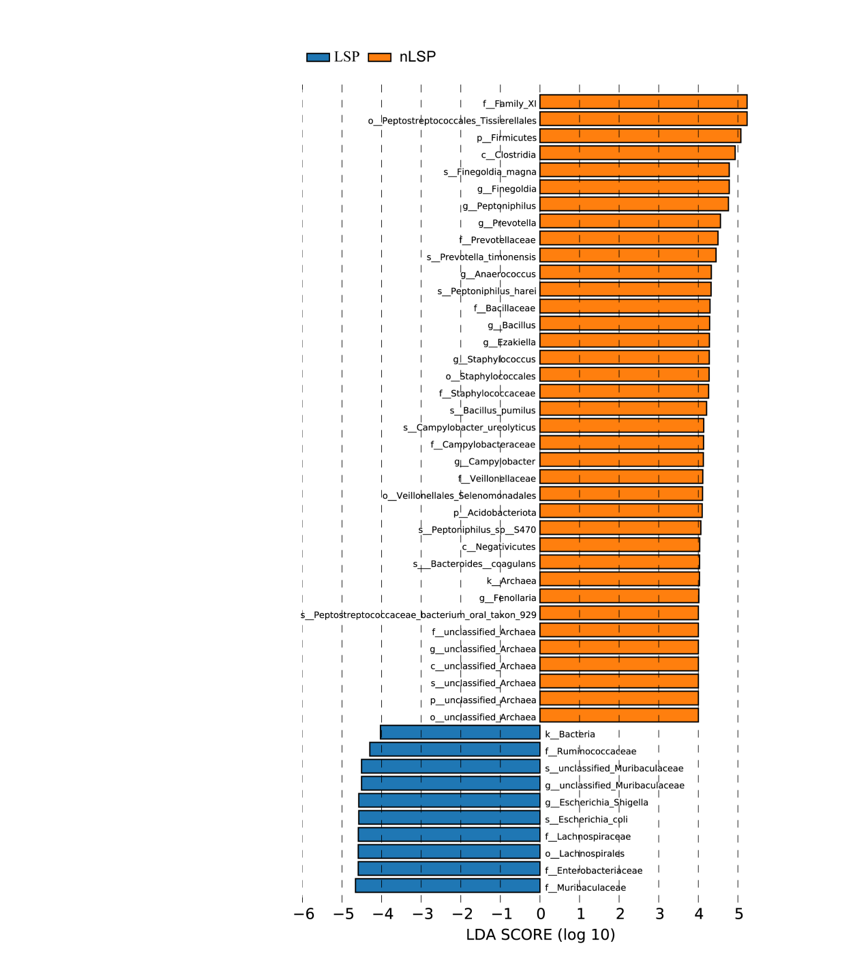
**

**Fig. S2** LDA scores of differential microbes between the MGLSc group and the control group.

**
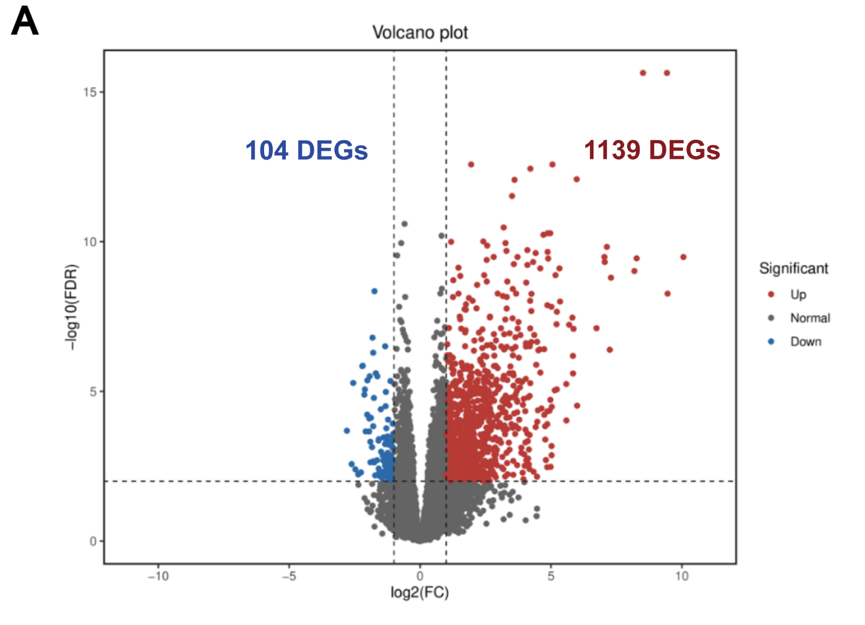
**

**Fig. S3** Volcano plot of up-regulated and down-regulated DEGs.


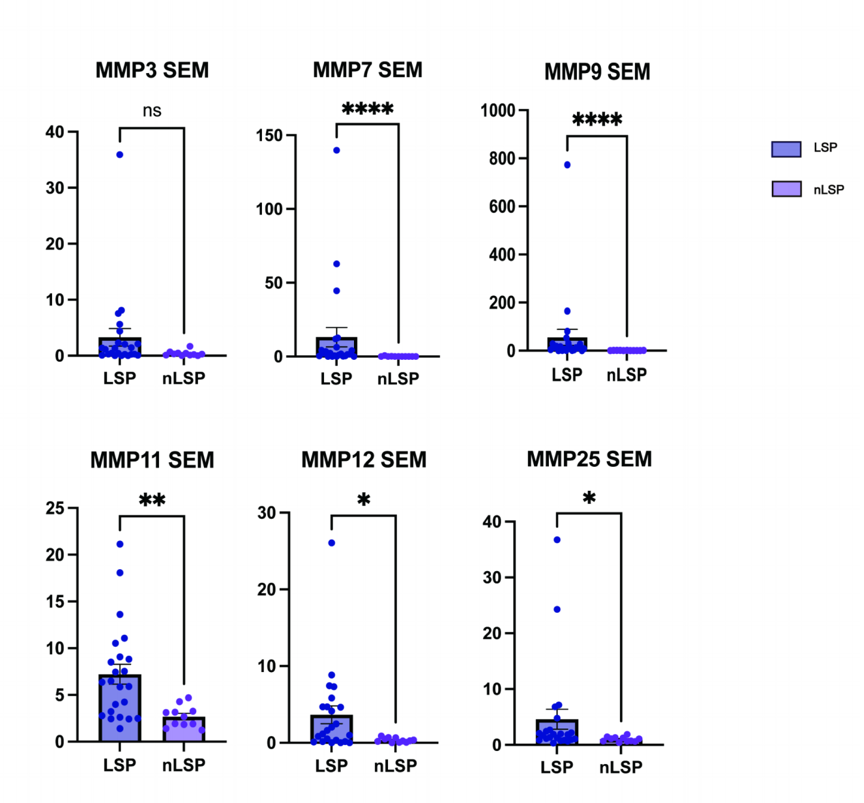


**Fig. S4.** Differential expression of MMP family genes in the MGLSc and Control Cohorts. The bars in the bar chart represent the mean of the data, and the error bars above or below the bars are based on the SEM (Standard Error of the Mean, SEM). * p < 0.05, ** p < 0.01.

**
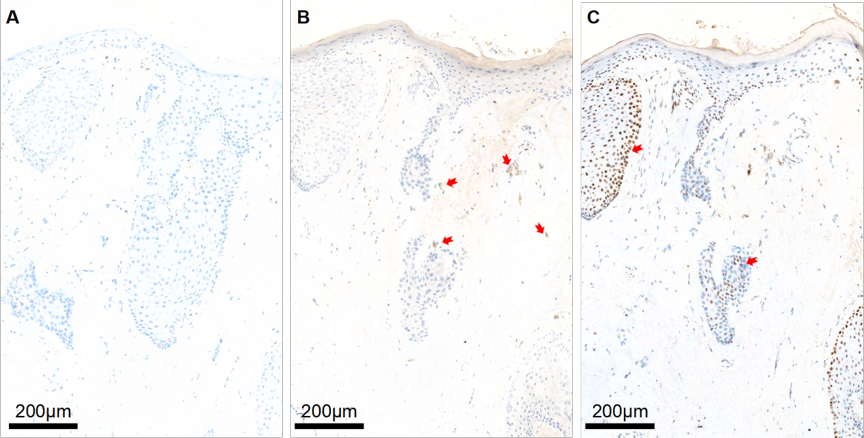
**

**Fig. S5.** Representative immunohistochemical staining of Gram-positive and Gram-negative bacterial markers in MGLSc preputial tissue. **(A)** Negative control staining of MGLSc tissue. **(B)** Lipoteichoic acid (LTA) staining representing Gram-positive bacterial components (7.7X). **(C)** Lipopolysaccharide (LPS) staining representing Gram-negative bacterial components (7.7X). Red arrows indicate positively stained areas.
